# Patient reported outcomes: past, present, and future

**DOI:** 10.1186/1745-6215-12-S1-A63

**Published:** 2011-12-13

**Authors:** Jane A Scott

**Affiliations:** 1 Global Strategic Marketing & Market Access, Janssen Global Services, High Wycombe, Buckinghamshire, HP12 4DP, UK

## 

For decades, clinical trials have relied on information from patients about their symptoms, functioning, and treatment experiences to evaluate treatment safety and efficacy. Today, information reported directly by patients (patient reported outcomes or “PROs”) using various technologies serves as the primary source of data about symptoms, functioning, health events, and the impact diseases and treatments have on the lives of patients and their families. The methods used to record and process that information has evolved over time so that today regulators require that trials use rigorous methods for all subjective assessments employed to evaluate treatments. This presentation is a brief review of the past, current, and future direction of PRO research and its implication for clinical trial measurement from the perspective of a PRO researcher working in medical product development trials.

